# Group-Level Neural Responses to Service-to-Service Brand Extension

**DOI:** 10.3389/fnins.2019.00676

**Published:** 2019-06-28

**Authors:** Taeyang Yang, Sung-Phil Kim

**Affiliations:** Brain-Computer Interface Laboratory, Department of Human Factors Engineering, Ulsan National Institute of Science and Technology, Ulsan, South Korea

**Keywords:** brand extension, event-related potential, P300, neuromarketing, EEG decoding

## Abstract

Brand extension is a marketing strategy leveraging well-established brand to promote new offerings provided as goods or service. The previous neurophysiological studies on goods-to-goods brand extension have proposed that categorization and semantic memory processes are involved in brand extension evaluation. However, it is unknown whether these same processes also underlie service-to-service brand extension. The present study, therefore, aims to investigate neural processes in consumers underlying their judgment of service-to-service brand extension. Specifically, we investigated human electroencephalographic responses to extended services that were commonly considered to fit well or badly with parent brand among consumers. For this purpose, we proposed a new stimulus grouping method to find commonly acceptable or unacceptable service extensions. In the experiment, participants reported the acceptability of 56 brand extension pairs, consisting of parent brand name (S1) and extended service name (S2). From individual acceptability responses, we assigned each pair to one of the three fit levels: high- (i.e., highly acceptable), low-, and mid-fit. Next, we selected stimuli that received high/low-fit evaluations from a majority of participants (i.e., >85%) and assigned them to a high/low *population-fit* group. A comparison of event-related potentials (ERPs) between population-fit groups through a paired *t*-test showed significant differences in the fronto-central N2 and fronto-parietal P300 amplitudes. We further evaluated inter-subject variability of these ERP components by a decoding analysis that classified N2 and/or P300 amplitudes into a high, or low population-fit class using a support vector machine. Leave-one-subject-out validation revealed classification accuracy of 60.35% with N2 amplitudes, 78.95% with P300, and 73.68% with both, indicating a relatively high inter-subject variability of N2 but low for P300. This validation showed that fronto-parietal P300 reflected neural processes more consistent across subjects in service-to-service brand extension. We further observed that the left frontal P300 amplitude was increased as fit-level increased across stimuli, indicating a semantic retrieval process to evaluate a semantic link between S1 and S2. Parietal P300 showed a higher amplitude in the high population-fit group, reflecting a similarity-based categorization process. In sum, our results suggest that service-to-service brand extension evaluation may share similar neural processes with goods-to-goods brand extension.

## Introduction

Brand rises as one of the key concepts in the contemporary marketing as consumers become increasingly exposed to a variety of brands, which affect their current and future purchase behavior. Brand extension is a marketing strategy that utilizes well-established brand names for new offerings (Loken and John, 1993). The offering is generally provided in a form of goods or services, which possess clearly distinct characteristics. Compared to goods offering, service offering portrays characteristics of inseparability of production and consumption, heterogeneity (i.e., difficulty to support consistent quality for individual consumers), intangibility, perishability, and a lack of ownership (Zeithaml et al., 1985; Iacobucci, 1998; Lovelock and Gummesson, 2004). Often, these characteristics make it more difficult for consumers to categorize service offerings rather than goods offerings.

To understand how consumers recognize, categorize, and evaluate brand extension, a number of cognitive neuroscience studies have investigated behavioral and neural responses to brand extension (Ma et al., 2007, 2008, 2010, 2014; Wang et al., 2012; Jin et al., 2015; Fudali-Czyż et al., 2016; Shang et al., 2017; Yang et al., 2018). In particular, a series of event-related potential (ERP) studies have suggested that a predominant cognitive process during brand extension evaluation is to compare a given product’s attributes to the corresponding attributes in brand memory. This process seemingly elicited ERP waveforms such as N270 (Ma et al., 2007) or P300 (Ma et al., 2008). Other investigations demonstrated the effect of emotions (Ma et al., 2010), unconscious categorization processing (Wang et al., 2012), and a stimuli presentation scheme (Ma et al., 2014) on neural responses to brand extension. Recent research has targeted broader aspects of brand extension by looking into brand extension strategies for launching a new brand (Jin et al., 2015), cultural differences (Fudali-Czyż et al., 2016) and the effect of logos (Shang et al., 2017). However, all of these studies have focused only on goods-to-goods brand extension. Because of the distinction in characteristics of service offerings (e.g., heterogeneity and intangibility), it is likely that a different cognitive processing would be involved in the evaluation of service-to-service brand extension.

Accordingly, Yang et al. (2018) recently investigated neural correlates of service-to-service brand extension evaluation. To study neural responses to brand extension, it is often necessary to provide brand extension stimuli with varying levels of fit between parent brand and extended offering. While fit levels between brand and goods offering can be objectively determined by the choice of the category of goods, it is more challenging to determine fit levels for service extension due to a difficulty in categorizing service offering. To address this issue, the investigators proposed a data-driven individual stimuli grouping method in which stimuli were grouped based on participant’s own behavioral response. As a result, high-fit and low-fit groups of stimuli varied across individual subjects. The ERP results showed that the frontal P300 (Novelty P3a) amplitude was higher for the low-fit than high-fit groups, suggesting that consumers recognized a low-fit service extension as a target stimulus in the evaluation task and evaluated service-to-service brand extension based on improbability, not approvability. That is, the subjects evaluated a given brand extension stimulus based on its level of improbability. However, the individual stimuli grouping method proposed in this previous study could not show participants’ neural response to common brand extension stimuli (for example, see Figure 2). Nonetheless, brand marketers may pursue to understand common response in a population of consumers when they evaluate a specific service-to-service brand extension.

Therefore, in the present study, we aim to find neural responses on stimuli commonly considered as high/low-fit to the population. For this purpose, we suggest a new stimuli grouping method. This method categorizes a stimulus as a high population-fit group if the acceptability across participants are universally high, a low population-fit group if those are universally low, or undetermined if mixed. In doing so, we can find neural responses of all participants to the same stimuli. In addition, we conduct a decoding analysis by estimating a population-fit level from the ERP amplitudes in order to demonstrate that which ERPs components indeed represent common neural responses across subjects. For this purpose, we build and train a classifier by using the data of subjects and then apply the trained classifier to the data of a novel subject to examine whether the classifier could accurately classify ERPs of the novel subject – labeled as the leave-one-subject-out cross validation. If the classifier could successfully estimate the population-fit level for a novel subject, it would mean that feature set of the classifier represent common neural responses across subjects.

## Materials and Methods

### Participants and Materials

In the experiment, 37 university students without any neurological disorders have participated, and they were prohibited to smoke or drink for a day before the experiment (18 Female, mean age 22.1 ± 0.33 years) (Yang et al., 2018). This study was carried out in accordance with the recommendations of Institutional Review Board of the Ulsan National Institute of Science and Technology (UNISTIRB-16-29-G) with written informed consent from all subjects. All subjects gave written informed consent in accordance with the Declaration of Helsinki. The protocol was approved by the Institutional Review Board of the Ulsan National Institute of Science and Technology. Among 37 participants, the data of one participant whose experiment was interrupted and four participants who did not answer the survey questions correctly were excluded from the analysis. In addition, the data of 13 participants were additionally excluded during the EEG analysis due to following issues: (1) the EEG data of four participants contained too much noise despite artifact reduction using the independent component analysis (ICA) method; and (2) the AR data of nine participants failed to give the minimum number of trials for each fit group (the minimum of 12 trials). Consequently, the EEG and behavioral data of a total of 19 participants were analyzed (nine males, mean age of 20.6 ± 0.48 years old).

**FIGURE 1:**
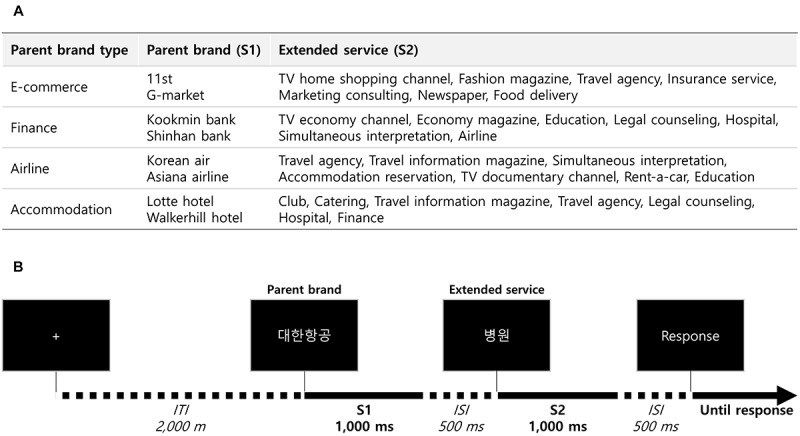
Experimental stimuli and task. **(A)** Experimental stimuli. A total of 56 stimuli pairs were pre-determined. **(B)** Experimental task. Participants were given successive brand extension stimuli including a parent brand name and extended service name, then asked to respond whether that brand extension is acceptable or not.

Fifty-six experimental stimuli consisting of eight parent brand names (S1) and seven extended service names per brand (S2) were collected in previous service brand extension studies (Lei et al., 2004; Brown et al., 2011; Arslan and Altuna, 2012). Specifically, we first determined four service categories (E-commerce, finance, airline, and accommodation) and then selected two popular brands from each of four categories. From *post hoc* interviews, we confirmed that participants were familiar to all of the selected brands and services. Extended services varied according to service category (see Figure 1A).

### Experimental Task

The S1–S2 paradigm with an explicit task, which has been used for previous brand extension studies (Ma et al., 2007, 2008), was adopted in our experiment (Figure 1B). In a single trial of the task, one of the eight parent brand names (S1) was presented, followed by one of the seven corresponding extended service names (S2). After the presentation of S2, participants were asked to respond using a keyboard (with their right hand) whether the provided extension was acceptable or not with a binary response (i.e., yes or no). Stimulus presentation time, inter-stimulus-interval (ISI) between S1 and S2, and inter-trial-interval (ITI) were set to 1,000, 500, and 2,000 ms, respectively. All the combinations of S1–S2 were randomly provided in a block of trials so that there were 56 trials performed per block. The experiment included one training block followed by four test blocks with break times (∼30 s on average) between blocks. As a result, each S1–S2 combination was repeatedly presented four times. At the end of the experiment, participants were asked to answer seven questions for each of the following brand extensions: acceptance for brand extension, expected quality to the extended service, preference to the extended service, similarity between service of parent brand and extended service, attitude toward brand extension, attitude toward parent brand, and involvement toward extended service.

### EEG Recordings

The experiment was conducted in a dim and electrically shielded room. The visual stimuli showing brand names or extended services were displayed on a 27-inch monitor (QH2700-IPSMS, Achieva Korea, Incheon, Korea) positioned at an approximately 60-cm distance from participants’ eyes. While participants performed the task, their scalp electroencephalography (EEG) signals were measured (band-pass filtering: 0.05–100 Hz, sampling rate: 500 Hz) using a 31-channel wet-electrode EEG recording system (actiCHamp, Brain products GmbH, Gliching, Germany) at the following electrode locations: FP1, FPz, FP2, F7, F3, Fz, F4, F8, FC9, FC5, FC1, FC2, FC6, FC10, T7, C3, Cz, C4, T8, CP5, CP1, CP2, CP6, P7, P3, Pz, P4, P8, O1, Oz, and O2 (in accordance with the International 10/20 system). An additional electrode was applied to the left mastoid (TP9) as a ground. The EEG signals were on-line referenced to the right mastoid (TP10). Impedance of every electrode was maintained below 10 kΩ during the recordings.

### Stimuli Grouping Method

**FIGURE 2:**
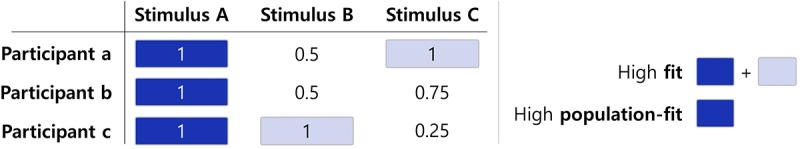
An illustration of a difference between the stimuli grouping methods for fit level and population-fit level. Fit level derives from an acceptance rate of one participant, while population-fit level is a result of acceptance rates (AR) of participants. In the figure, for example, the numerical value indicates an acceptance rate of stimulus from each participant. Each stimulus is labeled as high (AR = 1) or low (AR = 0), and mid (the other) fit level. Stimulus C is considered as high-fit level only for participant a. On the contrary, stimulus A is considered as high-fit for most of participants. Therefore, stimulus A is also considered as high population-fit level.

**FIGURE 3:**
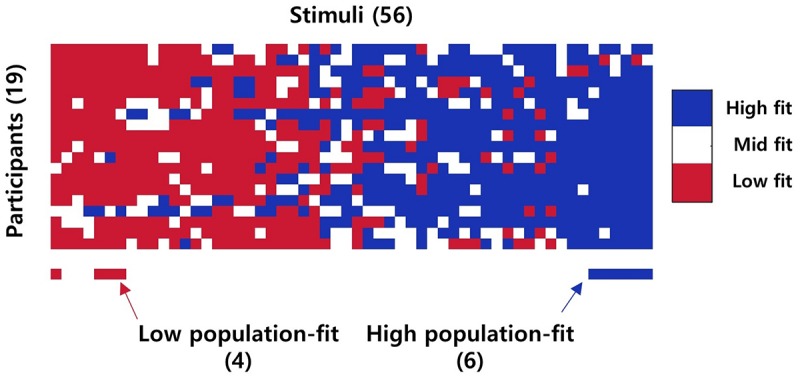
Stimuli grouping result. The image above indicates the fit level of each stimulus for each participant. The red bar below indicates low population-fit stimuli, while the blue bar indicates high population-fit stimuli. Only stimuli which was considered as high/low fit level for more than 85% of participants were selected as high/low population-fit stimuli.

In the previous study (Yang et al., 2018), authors compared ERPs between high and low-fit stimuli. However, this analysis has limitations in that the result is response to different stimulus among participants. Therefore, we wanted to show neural response to common stimuli among participants. As a result, we developed the term “*population-fit level*” from “fit level.” First, we calculated an acceptance rate (AR) by averaging four behavioral response (1 or 0). In next, we grouped stimuli in low (AR = 0), high (AR = 1), and mid (the others) according to AR score by participant. Finally, we collected only stimuli which was assigned to high/low fit for most of participants (>85% of participants in our case) and named them as high/low population-fit groups (see Figure 2). This stimuli grouping method yielded a low population-fit set with four stimuli (i.e., Kookmin Bank – hospital, Shinhan Bank – hospital, Shinhan Bank – airline, and Lotte Hotel – legal counseling) and a high population-fit set with six stimuli (i.e., Shinhan Bank – economy magazine, Korean Air – travel information magazine, Korean Air – simultaneous interpretation, Korean Air – TV documentary channel, Asiana Airline – simultaneous interpretation, and Asiana Airline – TV documentary channel; Figure 3).

### Data Analysis

The behavioral data acquired in the experiment besides acceptance of brand extension included reaction time (RT) and responses to seven questions after the experiment. We compared RT and survey responses between two sets of population-fit stimuli using a paired *t*-test (*N* = 19).

The recorded EEG signals were first filtered with 0.5 and 50-Hz cutoff frequencies using a FIR filter. Next, eye blinks and muscle artifacts were removed using the ICA method with visual inspection. Then, EEG epochs were extracted from a 1,250 ms data segment (−250 ∼ 1,000 ms) time-locked to the onset of the second stimulus (S2) and corrected to each baseline (i.e., −250 ∼ 0 ms time-locked to the onset of S2). ERP waveforms of high or low population-fit groups were obtained by averaging EEG data over the epochs of all the six stimuli (high population-fit) or four stimuli (low population-fit), for each channel and each participant. To evaluate statistical differences in ERPs between the population-fit groups, mean amplitudes of N2 (170 ∼ 230 ms time-locked to the onset of S2) and P300 ERP components (270 ∼ 330 ms time-locked to the onset of S2) were compared between the high- and low population-fit groups using a paired *t*-test (*N* = 19), at each EEG channel.

### Decoding Analysis

To find key component to describe neural responses to service-to-service brand extension, we conducted a decoding analysis with ERP waveforms. There have been many studies that conducted decoding analyses with a single-trial ERP waveform (e.g., Simanova et al., 2010; Li et al., 2012). However, our decoding analysis was different from these previous studies as it was designed to predict the population-fit level (i.e., either high- or low-fit) by building a classifier using selected EEG features from grand average ERP waveforms. We first extracted the feature of the mean amplitudes of N2 and P300, respectively, at every channel. Then, we selected channels which showed a significant difference in these amplitudes between the high and low population-fit groups (paired *t*-test, *p* < 0.05). This yielded seven channels for N2 and 10 for P300, respectively (see section “Decoding Results”). Next, we created a feature set of N2 (i.e., 7-dimensional feature vector) and that of P300 (i.e., 10-dimensional feature vector) individually, and fed each set to a decoding algorithm in order to examine which component provided better features for decoding. To verify the effect of dimensionality of a feature space, we also created a higher dimensional feature vector by combining both N2 and P300 features into a new feature set, resulting in an additional 17-dimensional feature vector. To evaluate decoding accuracy, a leave-one-subject-out cross validation scheme was used: among data from nineteen participants, we trained the classifier using those of eighteen participants and predicted the population-fit with those of the remaining participant, which was repeated nineteen times. The linear kernel support vector machine (SVM) was used as a classifier model.

## Results

### Behavioral Results

**FIGURE 4:**
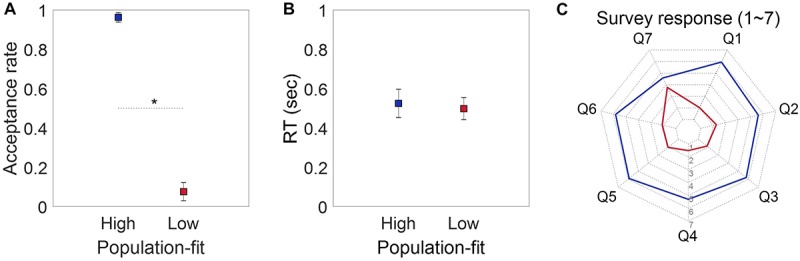
Behavioral results. **(A)** Population-wise acceptance rate of participants. **(B)** Population-wise reaction time of participants when evaluating whether a given brand extension was acceptable or not by pressing a keyboard. **(C)** Population-wise survey responses for seven questions. Paired *t*-test showed significant difference between two groups for every survey category.

A paired *t*-test showed a significant difference in the fit evaluation responses to the high [mean (*M*) = 0.963, *SE* = 0.0242] and low (*M* = 0.0757, *SE* = 0.0462) population-fit stimulus sets (*t*_(18)_ = 18.062, *p* < 0.001; Figure 4A). A paired *t*-test for the RT showed no difference between the high (*M* = 0.525 s, *SE* = 0.0722) and low (*M* = 0.498 s, *SE* = 0.0561) population-fit sets (*t*_(18)_ = 0.547, *p* = 0.591; Figure 4B). On the contrary, paired *t*-tests for each of the seven survey responses showed significant differences between the high and low population-fit sets for all questions (*ps* < 0.05; Figure 4C).

### ERP Results

**FIGURE 5:**
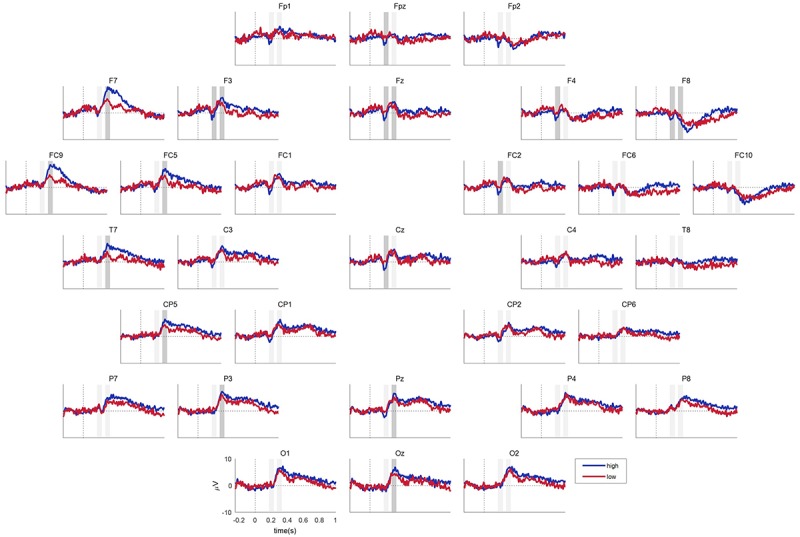
Event-related potential results. Blue and red lines indicate responses to the high and low population-fit stimulus sets, respectively. Gray boxes indicate time segments for N2 and P300 (from left to right), among which darker gray boxes denote a significant difference in the mean amplitudes between the high and low population-fit sets.

**FIGURE 6:**
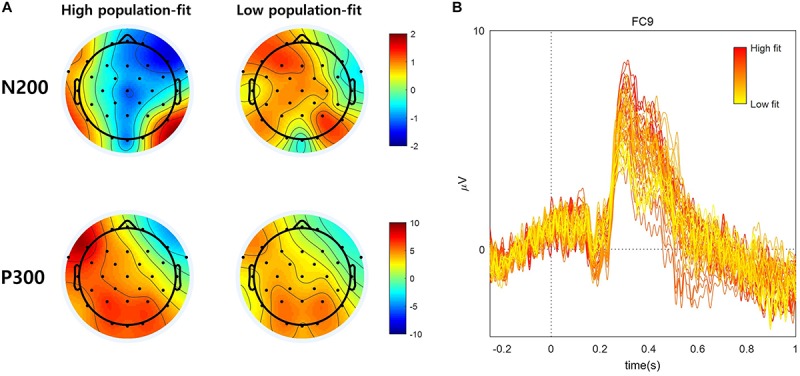
**(A)** EEG topographic map of high and low population-fit stimuli during N200 (170–230 ms) and P300 (270–330 ms). **(B)** Fifty ERP subsets according to the ratio of high-fit response at the left frontal area (i.e., FC9).

The ERPs obtained from the present study showed the prominent waveform of the N2 (170 ∼ 230 ms) ERP component over the fronto-central area and the P300 (270 ∼ 330 ms) ERP component over all scalp locations except for the right fronto-central area (Figure 5). We further compared a spatial pattern of the mean N2 and P300 amplitudes between the high and low population-fit sets using topographic maps (Figure 6A). It was shown that the mean N2 amplitudes at fronto-central channels were more negative in response to high population-fit stimuli than low population-fit stimuli. The mean P300 amplitudes were higher over left fronto-parietal channels in response to high population-fit stimuli than low population-fit stimuli. Additionally, we sorted stimuli according to the ratio of high-fit response and grouped each six stimuli by shifting the window, resulting in 50 stimulus subsets and corresponding ERPs (Figure 6B). These ERPs showed that the left frontal P300 amplitudes tended to gradually increase from low population-fit to high population-fit stimulus subsets.

To quantify the observations, a paired *t*-test was conducted for each EEG channel data. It revealed significant differences in the mean N2 amplitude between the high and low population-fit sets at FPz, F3, Fz, F4, F8, FC2, and Cz (Supplementary Table 1). In addition, the test showed significant differences in the mean P300 amplitude between the two population-fit sets at F7, F3, Fz, F8, FC9, FC5, T7, Cp5, P3, Pz, and Oz (Supplementary Table 2).

### Decoding Results

**FIGURE 7:**
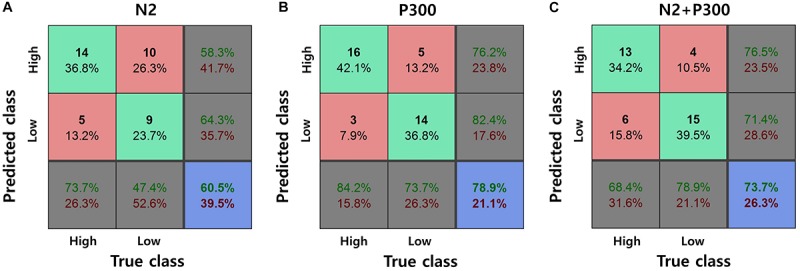
Classification result with the selected ERP features at channels of interests. **(A)** Mean N2 amplitude. **(B)** Mean P300 amplitude. **(C)** Both mean N2 and P300 amplitudes.

Features of the mean N2 amplitude were extracted from ERP waveforms at seven electrodes (i.e., FPz, F3, Fz, F4, F8, FC2, and Cz). Features of the mean P300 amplitude were extracted from ERP waveforms at ten electrodes (i.e., F7, F3, Fz, FC9, FC5, T7, CP5, P3, Pz, and Oz). The decoding analysis resulted in the classification accuracy of 78.95% when using the mean P300 amplitude feature set, 60.53% when using the mean N2 amplitude feature set, and 73.68% when using the combined feature set (Figure 7). Note that the chance level was 50% as we extracted features from a single ERP waveform for each class (i.e., high and low population-fit classes) in each participant.

## Discussion

A previous study investigated service-to-service brand extension and its neural substrates (Yang et al., 2018). However, this previous study grouped stimuli based on the subjective evaluation of a fit level, leading that the analyzed neural activities of individual participants were responses to different stimuli. In contrast, the present study suggested a new stimuli grouping method to create the same set of stimuli based on the ratio of participants’ common evaluation (i.e., population-fit level). As such, we could analyze the neural responses to identical stimuli across all participants.

The newly suggested grouping method did not have an effect on the behavioral result (Figure 4), whereas the EEG result was substantially affected by it. The remarkable difference in the ERP waveform was the spatial pattern of P300 and the absence of N400. In Yang et al. (2018), right frontal P300 and N400 amplitudes were prominent in response to high individual-fit brand extension stimuli. However, in the present study, fronto-central N2 and left frontal-parietal P300 amplitudes were prominent and significantly distinguished between high and low population-fits (Figure 5). Although there was also a significant difference of P300 amplitudes at the right frontal EEG channel (i.e., F8), right frontal P300 was not considered in further analysis because its amplitude level and waveform did not fit well to the standard definition of P300.

Our ERP result revealed that the mean N2 amplitudes at fronto-central channels (e.g., FPz, Fz, and Cz) were significantly different between population-fits, showing larger negative amplitudes for high population-fit stimuli. In the decoding analysis (Figure 7), the classification accuracy with the mean N2 amplitude was only 60.53%, whereas that with the mean P300 amplitude was 78.95%. This result indicates that N2 response was not consistent enough across subjects and not appropriate, whereas P300 is an appropriate ERP component to be a key component investigate the common response in consumers when evaluating service-to-service brand extension.

Ma et al. (2008) reported that parietal P300 was elicited in both high- and low-fit conditions but with a larger amplitude for the high-fit condition, indicating that categorizing processing could induce P300. They suggested that larger parietal P300 amplitudes might be associated with a larger degree of perceived similarity of attributes between parent brand’s goods and extended goods. Our study showed the same result at the parietal area, which depicts that there is also the similarity-based categorizing processing in service-to-service brand extension evaluation. In our result, the parietal P300 showed dominance in the left hemisphere. This pattern might be related to the activity of the left anterior temporal pole (Brodmann area 38), which is shown to be more active with complete sentences than scrambled ones, playing a potential role in the composition of sentence meaning (Vandenberghe et al., 2002). Therefore participants might understand a relationship between S1 and S2 more naturally for high population-fit stimuli, akin to the composition of a sentence.

**FIGURE 8:**
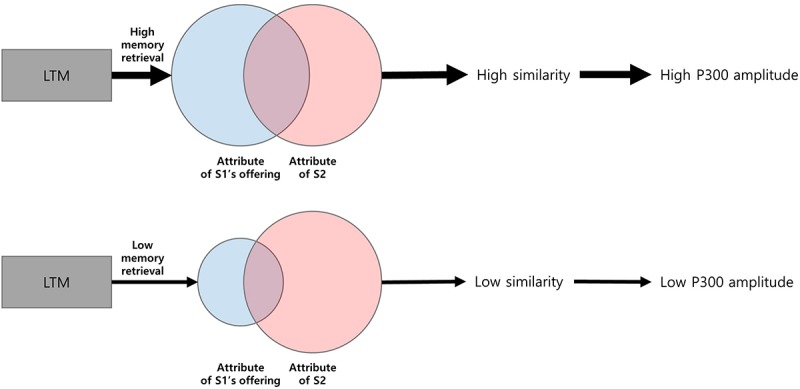
Conceptual diagram of memory-categorizing processing.

Our results also revealed the left dominance of P300 amplitudes at the frontal area. This spatial pattern was more remarkable for the high population-fit condition (Figure 6A). In addition, the P300 amplitude at the left frontal area (i.e., FC9) showed a trend that gradually increases along the ratio of high-fit response (Figure 6B). According to previous research, Brodmann area 47, which is close to the left frontal area, is related to language-related semantic retrieval processing (Zhang et al., 2004; Lehtonen et al., 2005) Therefore, left frontal dominance of P300 amplitudes might be related to semantic retrieval processing during the evaluation of brand extension. Overall, we conceptualize a two-stage brand extension evaluation model as follows: in the first stage, memory retrieval of parent brand elicits attributes of parent brand’s offering; and in the second stage, similarity of attributes between brand’s offering and S2 is perceived (Figure 8). If brand extension successfully retrieves abundant attributes of parent brand’s offering, it would be more likely that those attributes and the attributes of S2 will become more similar, which is reflected by larger P300 amplitudes. Although Ma et al. (2008) suggested a similar categorization model, our study could extend the scope of models from only goods-to-goods brand extension to including service-to-service brand extension.

Our result that high population-fit stimuli revealed higher frontal P300 amplitude provides us background knowledge to extract significant features to predict brand extension fit levels using EEG. The prediction is meaningful in that it helps marketers know consumers’ attitude toward the brand extension before launching. However, in most real situations, marketers would want to know the fit level of a specific brand. Despite diverse analyses, this study yet conducted a group-wise fit-level analysis, rather than the individual stimulus-level. Therefore, in extended research in the future, the experiment should be designed to consider the stimulus-level analysis. In addition, to overcome the limitation that the connection between our results and some references are not strong due to a difference in apparatus, functional magnetic resonance imaging (fMRI) should be used to investigate the evaluating process of brand extension.

## Conclusion

The present study investigated common neural processes in a group of people when they evaluated service-to-service brand extension. To find common neural responses, we applied a new stimuli grouping method in which the high-, or low-fit brand extension stimuli were selected based on population-fit, not individual evaluations of fit levels. The analysis of ERPs in response to the high- and low-population fit stimuli showed a significant difference in left fronto-parietal P300 amplitudes between population-fit stimulus groups where the P300 amplitude was higher for the high population-fit group. In addition, the P300 amplitude tended to increase as a fit level increased. A decoding analysis showed the low inter-subject variability of the P300 amplitude, demonstrating that we could decode the fit level from the P300 amplitude of one subject using a classifier trained using the data of all other subjects. Our results suggest that left fronto-parietal P300 may provide neural evidence for the acceptability of a new service-to-service brand extension to a population of consumers, which may involve semantic memory retrieval and similarity-based categorization.

## Data Availability

The datasets generated for this study are available on request to the corresponding author.

## Ethics Statement

This study was carried out in accordance with the recommendations of Institutional Review Board of the Ulsan National Institute of Science and Technology (UNISTIRB-16-29-G) with written informed consent from all subjects. All subjects gave written informed consent in accordance with the Declaration of Helsinki. The protocol was approved by the Institutional Review Board of the Ulsan National Institute of Science and Technology.

## Author Contributions

TY participated in all aspects of the work, designed and conducted the experiment, analyzed the data, and wrote the manuscript. S-PK oversaw the study and managed every part of the research. Both authors read and approved the final manuscript.

## Conflict of Interest Statement

The authors declare that the research was conducted in the absence of any commercial or financial relationships that could be construed as a potential conflict of interest.
