# Supplementary material for: Group-Level Neural Responses to Service-to-Service Brand Extension
Source: Front Neurosci. 2019 Jun 28;13:676. doi: 10.3389/fnins.2019.00676 (PMC6610219; doi:10.3389/fnins.2019.00676)
Supplement: Supplementary file 1 [file Table_1.docx]

Supplementary Material

Group-level neural responses to service-to-service brand extension

Taeyang Yang, Sung-Phil Kim*

*** Correspondence:** Sung-Phil Kim: [spkim@unist.ac.kr](mailto:spkim@unist.ac.kr)

Supplementary Table 1. The mean N2 amplitudes of ERP, standard deviation in parentheses and the result of paired t-test at different electrode sites. Bolded contents indicate statistically significant difference (*p* < 0.05)

| **Electrode site** | **High-group fit (μV)** | **Low-group fit (μV)** | ***t*_(18)_ value** | ***p* value** |
| --- | --- | --- | --- | --- |
| Fp1 | -0.485 (0.727) | 1.005 (0.840) | -1.611 | 0.124 |
| **Fpz** | **-1.010 (0.798)** | **0.673 (0.692)** | **-2.384** | **0.028** |
| Fp2 | -1.199 (0.968) | 0.246 (0.624) | -1.629 | 0.121 |
| F7 | 0.690 (0.600) | 1.132 (0.875) | -0.534 | 0.600 |
| **F3** | **-0.524 (0.633)** | **1.264 (0.766)** | **-2.492** | **0.023** |
| **Fz** | **-0.981 (0.606)** | **1.088 (0.812)** | **-2.643** | **0.017** |
| **F4** | **-1.294 (0.618)** | **0.434 (0.786)** | **-2.481** | **0.023** |
| **F8** | **-1.637 (0.605)** | **-0.209 (0.645)** | **-2.457** | **0.024** |
| FC9 | 0.840 (0.559) | 0.851 (0.675) | -0.015 | 0.988 |
| FC5 | 0.070 (0.598) | 0.776 (0.777) | -0.828 | 0.418 |
| FC1 | -0.574 (0.569) | 0.893 (0.842) | -1.822 | 0.085 |
| **FC2** | **-0.840 (0.638)** | **0.783 (0.790)** | **-2.106** | **0.050** |
| FC6 | -0.995 (0.611) | -0.237 (0.612) | -0.968 | 0.346 |
| FC10 | -0.895 (0.477) | -0.597 (0.475) | -0.573 | 0.574 |
| T7 | 0.661 (0.474) | 0.354 (0.689) | 0.422 | 0.678 |
| C3 | -0.389 (0.480) | 0.843 (0.815) | -1.539 | 0.141 |
| **Cz** | **-1.215 (0.598)** | **0.939 (0.840)** | **-2.305** | **0.033** |
| C4 | -0.793 (0.545) | 0.809 (0.865) | -1.704 | 0.106 |
| T8 | -0.319 (0.530) | -0.276 (0.539) | -0.081 | 0.937 |
| CP5 | 0.150 (0.518) | 0.797 (0.773) | -0.858 | 0.402 |
| CP1 | -0.664 (0.521) | 0.902 (0.774) | -1.947 | 0.067 |
| CP2 | -0.906 (0.511) | 0.609 (0.683) | -1.790 | 0.090 |
| CP6 | -0.111 (0.445) | 0.263 (0.561) | -0.537 | 0.598 |
| P7 | 0.814 (0.688) | 0.974 (0.770) | -0.199 | 0.844 |
| P3 | -0.292 (0.769) | 0.742 (0.848) | -1.149 | 0.266 |
| Pz | -0.967 (0.634) | 0.549 (0.715) | -1.794 | 0.090 |
| P4 | 0.039 (0.589) | 1.240 (0.658) | -1.536 | 0.142 |
| P8 | 1.308 (0.481) | 0.933 (0.551) | 0.610 | 0.550 |
| O1 | -0.202 (0.948) | 0.370 (0.716) | -0.619 | 0.544 |
| Oz | -1.079 (0.823) | -0.395 (0.613) | -0.937 | 0.361 |
| O2 | 0.219 (0.712) | 0.705 (0.654) | -0.787 | 0.441 |

Supplementary Table 2. The mean P300 amplitudes of ERP, standard deviation in parentheses and the result of paired t-test at different electrode sites. Bolded contents indicate statistically significant difference (*p* < 0.05)

| **Electrode site** | **High-group fit (μV)** | **Low-group fit (μV)** | ***t*_(18)_ value** | ***p* value** |
| --- | --- | --- | --- | --- |
| Fp1 | 3.598 (1.050) | 2.193 (0.970) | 1.272 | 0.220 |
| Fpz | 1.310 (1.154) | 0.535 (0.813) | 0.931 | 0.364 |
| Fp2 | -1.396 (1.477) | -0.589 (0.923) | -0.740 | 0.469 |
| **F7** | **8.760 (1.001)** | **4.576 (1.196)** | **4.776** | **0.000** |
| **F3** | **5.102 (0.887)** | **3.144 (0.855)** | **3.033** | **0.007** |
| **Fz** | **3.679 (0.742)** | **2.183 (0.788)** | **2.303** | **0.033** |
| F4 | 0.656 (0.810) | 0.939 (0.892) | -0.455 | 0.655 |
| **F8** | **-3.041 (0.920)** | **-1.460 (0.958)** | **-2.136** | **0.047** |
| **FC9** | **7.664 (1.077)** | **4.012 (0.987)** | **4.664** | **0.000** |
| **FC5** | **6.292 (0.878)** | **3.705 (0.880)** | **3.419** | **0.003** |
| FC1 | 4.559 (0.814) | 3.336 (0.802) | 1.978 | 0.063 |
| FC2 | 2.929 (0.731) | 2.419 (0.797) | 0.853 | 0.405 |
| FC6 | -0.029 (0.667) | 0.120 (0.684) | -0.235 | 0.817 |
| FC10 | -2.608 (0.698) | -1.552 (0.642) | -1.698 | 0.107 |
| **T7** | **5.749 (0.749)** | **2.699 (0.773)** | **4.732** | **0.000** |
| C3 | 5.178 (0.846) | 3.976 (0.783) | 1.646 | 0.117 |
| Cz | 4.033 (0.785) | 2.930 (0.853) | 1.345 | 0.195 |
| C4 | 2.883 (0.670) | 2.882 (0.826) | 0.001 | 0.999 |
| T8 | 0.202 (0.463) | -0.264 (0.513) | 1.160 | 0.261 |
| **CP5** | **5.593 (0.692)** | **3.827 (0.737)** | **2.904** | **0.009** |
| CP1 | 5.237 (0.700) | 3.938 (0.712) | 2.062 | 0.054 |
| CP2 | 4.240 (0.660) | 3.575 (0.659) | 1.074 | 0.297 |
| CP6 | 2.772 (0.449) | 2.597 (0.500) | 0.316 | 0.756 |
| P7 | 4.492 (0.844) | 2.956 (0.866) | 1.970 | 0.064 |
| **P3** | **6.572 (0.667)** | **5.133 (0.679)** | **2.211** | **0.040** |
| **Pz** | **5.648 (0.686)** | **4.295 (0.725)** | **2.580** | **0.019** |
| P4 | 5.840 (0.688) | 5.180 (0.581) | 1.123 | 0.276 |
| P8 | 3.985 (0.498) | 3.269 (0.479) | 1.464 | 0.161 |
| O1 | 6.192 (0.863) | 5.163 (0.684) | 1.311 | 0.206 |
| **Oz** | **5.635 (0.782)** | **4.048 (0.767)** | **2.219** | **0.040** |
| O2 | 6.165 (0.867) | 4.909 (0.765) | 1.881 | 0.076 |
